# Prolyl hydroxylase regulates axonal rewiring and motor recovery after traumatic brain injury

**DOI:** 10.1038/cddis.2015.5

**Published:** 2015-02-12

**Authors:** S Miyake, R Muramatsu, M Hamaguchi, T Yamashita

**Affiliations:** 1 Department of Molecular Neuroscience, Graduate school of Medicine, Osaka University, Suita, Osaka, Japan; 2 Japan Science and Technology Agency, Core Research for Evolutional Science and Technology, Chiyoda, Tokyo, Japan; 3 Precursory Research for Embryonic Science and Technology, Japan Science and Technology Agency, 5, Sanbancho, Chiyoda-ku, Tokyo, Japan

## Abstract

Prolyl 4-hydroxylases (PHDs; *PHD1, PHD2*, and *PHD3*) are a component of cellular oxygen sensors that regulate the adaptive response depending on the oxygen concentration stabilized by hypoxia/stress-regulated genes transcription. In normoxic condition, PHD2 is required to stabilize hypoxia inducible factors. Silencing of *PHD2* leads to the activation of intracellular signaling including RhoA and Rho-associated protein kinase (ROCK), which are key regulators of neurite growth. In this study, we determined that genetic or pharmacological inhibition of PHD2 in cultured cortical neurons prevents neurite elongation through a ROCK-dependent mechanism. We then explored the role of PHDs in axonal reorganization following a traumatic brain injury in adult mice. Unilateral destruction of motor cortex resulted in behavioral deficits due to disruption of the corticospinal tract (CST), a part of the descending motor pathway. In the spinal cord, sprouting of fibers from the intact side of the CST into the denervated side is thought to contribute to the recovery process following an injury. Intracortical infusion of PHD inhibitors into the intact side of the motor cortex abrogated spontaneous formation of CST collaterals and functional recovery after damage to the sensorimotor cortex. These findings suggest PHDs have an important role in the formation of compensatory axonal networks following an injury and may represent a new molecular target for the central nervous system disorders.

Damage to the adult central nervous system (CNS) leads to disruption of neural networks, thus causing significant impairment of neurological processes such as motor, sensory, and cognitive function. Over time, partial functional improvement in the neurological symptoms is sometimes observed. This is considered to be the result of the compensation by various forms of plasticity in the remnant neuronal network.^1, 2^ After a traumatic brain injury (TBI), cortical damage often causes motor deficits owing to the loss of descending motor pathways, including the corticospinal tract (CST), which connects cortical layer V neurons with their spinal targets. After an injury, new fibers sprout from neurons in the intact side of the corticospinal tract and extend into the denervated side at multiple levels of the brain and the spinal cord and form a spinal detour circuit that contributes to partial recovery of motor function.^3^ Defining the mechanisms underlying spontaneous restoration of the neuronal network remains an unresolved challenge; therefore, identification of the molecular basis of axonal regeneration and compensation may contribute to the development of new therapeutic strategies for the treatment of brain injury.

Cellular oxygen sensing pathways regulated by hypoxia-inducible factors (HIFs) are important mediators of the cellular injury response. HIFs are dimeric transcription factors comprised of an oxygen-sensitive HIF-*α* subunit and an oxygen-independent subunit, HIF-*β*. In high oxygen conditions, Prolyl 4-hydroxylases (PHDs: PHD1, PHD2, PHD3) hydroxylate key proline residues of HIF-*α*,^4^ which subsequently targets HIF-*α* for degradation by ubiquitin–ligase complexes.^5^ Each PHD differs in the relative abundance of their mRNA, but all the *PHD* mRNA show a ubiquitous pattern of expression that includes the brain.^6, 7^ The role of PHDs has been intensively studied in inflammation, tumor growth, metabolism, and hematopoetic stem cell residing in a hypoxic niche;^8, 9^ however, their role in the nervous system is largely unknown.

Axon navigation is regulated by attractive and repulsive cues from extracellular signals.^10^ Recently, it was reported that oxygen supply prevents the formation of aberrant axon projections, at least in part through maintenance of such guidance signals in *Caenorhabditis elegans*;^11^ thus, oxygen sensing and downstream signal transduction may be a regulator of axon guidance cues. In addition, suppression of *PHD2* enhances the activation and protein expression of the small GTPase RhoA,^12^ a key molecule inhibiting axon growth after CNS injury.^13^ We thus hypothesized that PHDs are involved in axon rewiring following a CNS injury. In this study, we show that PHD inhibitors prevent neurite elongation in cerebral cortical neurons *in vitro*. Expression of RhoA protein increased upon treatment with PHD inhibitors, resulting in suppression of neurite elongation. *In vivo*, we found that infusion PHD inhibitors into contralateral motor cortex attenuated CST sprouting and recovery of neurological deficits in a mouse model of TBI.

## Results

### PHD inhibitors prevent neurite elongation in cultured cortical neurons

**Figure 1:**
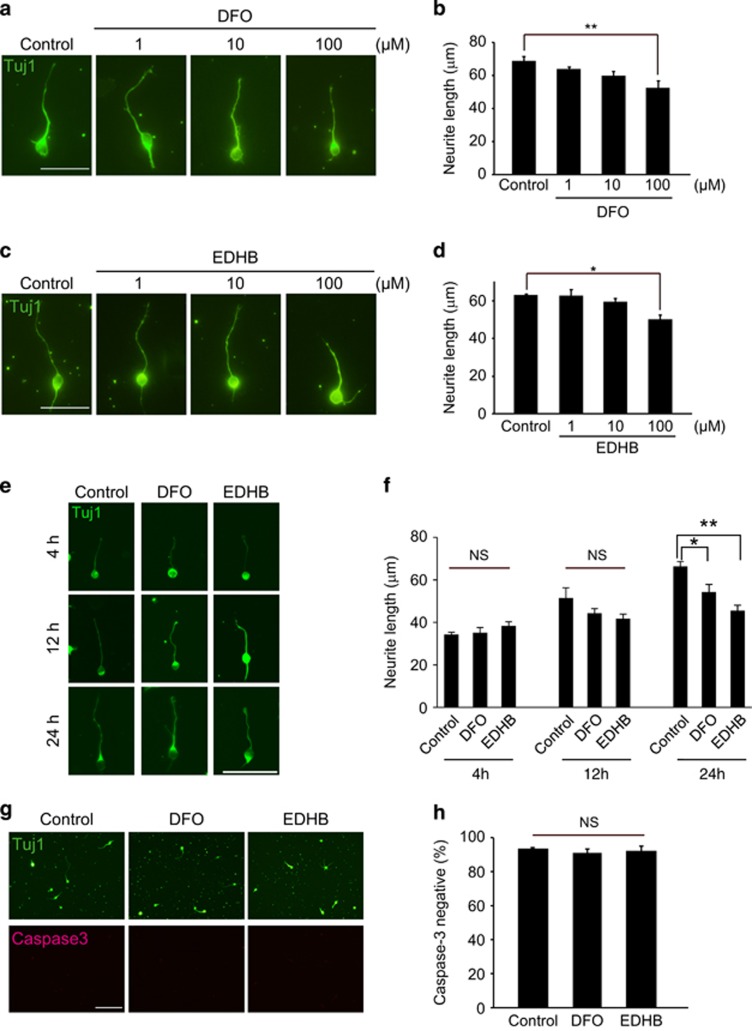
Treatment with PHD inhibitors prevents neurite outgrowth without affecting cell survival. (**a** and **c**) Representative images of cultured cortical neurons stained with an anti-class III *β*-tubulin (Tuj1) antibody (labeled with Alexa Fluor 488). Cortical neurons were treated with desferrioxamine (DFO) (**a**) or ethyl-3,4-dihydroxybenzoate (EDHB) (**c**) for 24 h and then stained with anti-Tuj1 antibody. The graphs (**b** and **d**) show mean±S.E.M.) of neurite length observed in Tuj1-positive neurons. (**e**) Representative images of the cultured cortical neurons stained with a Tuj1 antibody (labeled with Alexa Fluor 488). Cortical neurons were treated with DFO or EDHB for indicated periods and then stained with anti-Tuj1 antibody. The Graphs (**f**) show mean±S.E.M. of neurite length observed in Tuj1-positive neurons. (**g)** Representative images of cultured cortical neurons stained with anti-Tuj1 (labeled with Alexa Fluor 488) and anti-caspase 3 (labeled with Alexa Fluor 568). Cortical neurons were treated with DFO or EDHB for 24 h and then costained with anti-Tuj1and anti-caspase 3 antibodies. (**h)** The ratio of the number of cells double-positive for anti-caspase 3 and anti-Tuj1 immunohistochemical staining to the total number of Tuj1-positive cells. Values are represented as mean±S.E.M. (*n*=3-4). **P<0.01, *P<0.05 using one-way analysis of variance followed by Tukey's test. Scale bars, 50 *μ*m

We first tested the role of PHD-mediated signaling in neurite elongation. We prepared cultured neurons obtained from the cerebral cortex of P1 mice, and treated them with various concentrations of the PHD inhibitors desferrioxamine (DFO), an iron chelator, or ethyl-3,4-dihydroxybenzoate (EDHB). After 24 h, we measured the neurite length of the cultured neurons that were positive for Tuj1 (class III *β*-tubulin). Treatment with either PHD inhibitor-suppressed neurite elongation in a concentration-dependent manner (Figures 1a–d). Exposure of PHD inhibitors produced inhibition of neurite elongation in a time-dependent fashion and maximal effect was seen at 24 h (Figures 1e and f). These results suggest that PHDs mediate neurite elongation in cortical neurons.

As reported previously, PHDs provide neuroprotection against glutamate-induced toxicity in cultured cortical neurons.^14^ We checked the effects of PHD inhibitors on cell survival in cultured cortical neurons by culturing cortical neurons with or without PHD inhibitors for 24 h, and then counting the number of the cells that were double-positive for caspase-3 and Tuj1. There were no significant differences across all groups in the percentage caspase-3-positive/Tuj1-positive cells in the total number of Tuj1-positive cells, suggesting that PHD inhibitors did not affect cell viability (Figures 1g and h). These results suggest that PHD inhibitors prevent neurite elongation independently from cell survival.

### RhoA/ROCK pathway is required for the effect of PHD inhibitors on neurite elongation

**Figure 2:**
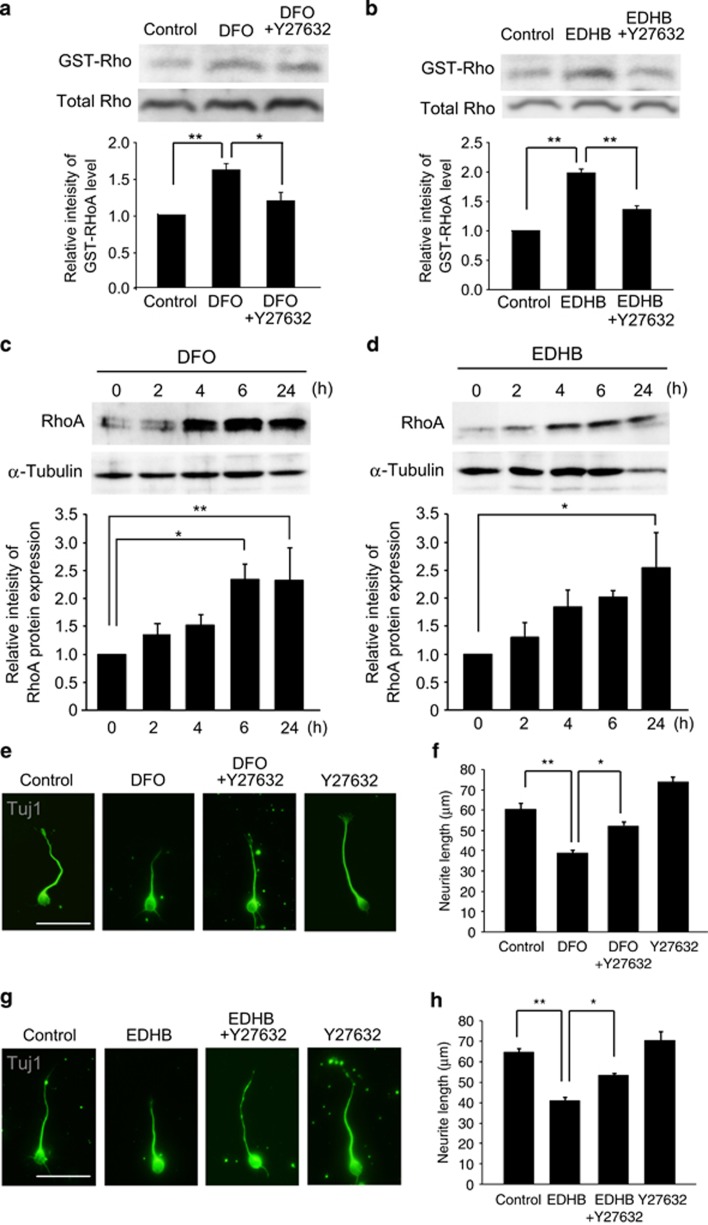
ROCK inhibition abrogates the suppressive effect of PHD inhibitors on neurite outgrowth. **(a** and **b**) Western blot analysis of GST-RhoA (top row) and total RhoA (bottom row). Relative level of RhoA activation in cortical neuron. Cortical neurons were pretreated with Y27632 (10 *μ*M) for 30 min, and then treated with DFO (**a**) or EDHB (**b**) for 5 min. Values are represented as mean±S.E.M. (*n*=3). (**c** and **d**) Western blot analysis of RhoA (top row) and α-tubulin (bottom row). Relative expression of RhoA in cortical neurons treated with DFO (**c**), or EDHB (**d**). Values are represented as mean±S.E.M. (*n*=5). (**c**, **e**, and **g**) Representative images of cultured cortical neurons stained with anti-Tuj1 antibody (labeled with Alexa Fluor 488). Cortical neurons were pretreated with Y27632 (10 *μ*M) for 30 min, and then treated with DFO (**e**) or EDHB (**g**) for 24 h. (**f** and **h**) Graphs show mean±S.E.M. of the neurite length of Tuj1-positive neurons (*n*=3). **P<0.01, *P<0.05 by using one-way analysis of variance followed by Tukey's test. Scale bar, 50 *μ*m

We next investigated molecular mechanisms of PHD inhibitor-suppressed neurite elongation, focusing on the role of RhoA signaling in neurite elongation regulated by PHD inhibitors, because PHD is known to be involved in RhoA-dependent cell morphology.^12^ To test this, we assessed whether neuronal effect of PHD inhibitors are dependent on the Rho activation. Rho activation was determined using the RhoA binding domain of the effector protein.^15^ After 5 min of stimulation, cell extracts contained increased amounts of GTP-RhoA compared with control (Figures 2a and b), suggesting that PHD inhibition enhances RhoA activation. We examined if RhoA activation by PHD inhibitors is canceled by treatment with Rho-associated kinase (ROCK) inhibitor. Treatment with Y27632, an inhibitor of ROCK, abolished PHD inhibitors-mediated increased amount of GTP-RhoA (Figures 2a and b). Moreover, we performed western blot analysis and found that treatment with DFO or EDHB resulted in gradual increase in the level of RhoA protein expression in cultured cortical neurons (Figures 2c and d). Thus, PHD inhibitors enhance RhoA protein expression in cortical neurons.

We then examined if blocking RhoA signaling could rescue PHD inhibitor-mediated suppression of neurite elongation. Treatment with Y27632, an inhibitor of Rho-associated kinase (ROCK), was found to restore the suppressive effect of PHD inhibitors on the neurite outgrowth (Figures 2e–h). Moreover, Y27632 by itself did not promote neurite elongation in the control group. Therefore, PHD inhibitors prevent cortical neurite elongation by a mechanism dependent on RhoA-ROCK pathway.

### Silencing of PHD2 inhibits neurite elongation by a mechanism dependent on ROCK

**Figure 3:**
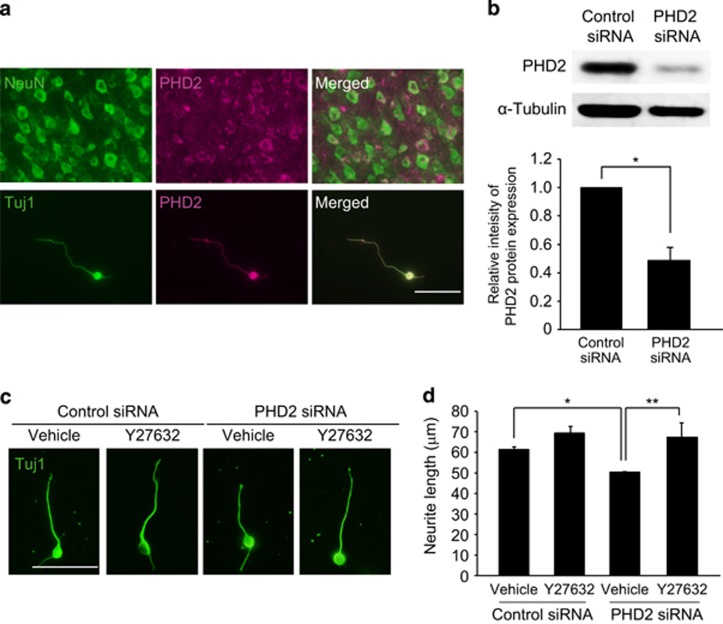
PHD2 regulates neurite outgrowth by a mechanism dependent on ROCK. (**a**) (top panels) Representative images of double-staining for PHD2 (labeled with Alexa Fluor 568) and NeuN (labeled with Alexa Fluor 488) in cerebral cortex of adult mice. (bottom panels) Representative images of double staining for PHD2 (labeled with Alexa Fluor 568) and Tuj1 (labeled with Alexa Fluor 568) in cultured cortical neurons. (**b**) Western blots showing the expression of PHD2 (top row) and *α*-tubulin (bottom row) in cultured cortical neurons transfected with PHD2 siRNA. The graph shows the relative expression of PHD2. Values are represented as mean±S.E.M. (*n*=4). (**c**) Representative images of cultured cortical neurons stained with anti-Tuj1 antibody (labeled with Alexa Fluor 488). Cortical neurons were transfected with PHD2 siRNA. After culture for 72 h, cells were replated and incubated for additional 24 h in the presence or absence of Y27632. (**d**) Graph shows mean±S.E.M. of neurite length of Tuj1-positive neuron. Graph shows mean±S.E.M. of neurite length of Tuj1-positive neurons. The cell number: **d** (*n*=126–150 neurons). **P<0.01, *P<0.05 using Student's *t*-test for **b**, and Scheffe's test for **d**. Scale bar, 50 *μ*m

Disturbances in oxygen availability due to the destruction of vasculature and impaired blood–brain barrier integrity at the lesion are implicated in the pathophysiology of the CNS disorders.^16^ Meanwhile, axonal plasticity and remodeling after CNS injury are observed at the distal part of lesion in which capillaries remain intact. Under normoxic condition, silencing of *PHD2* completely eliminates hydroxylation of HIF-1*α*, suggesting that *PHD1* and *PHD3* do not contribute to oxygen-regulated signal transduction.^17^ Thus, we explored the involvement of PHD2 in neurite elongation. We first carried out immunohistochemical analysis to investigate the expression of PHD2 in cortical neurons. Double staining by using anti-PHD2 and anti-Tuj1 antibodies showed that PHD2 was expressed in cultured cortical neuron (Figure 3a) and we further confirmed the expression of PHD2 in NeuN^+^ cells of layer 5 in adult cerebral cortex (Figure 3a).

To assess the role of PHD2 in neurite elongation, we inhibited PHD2 protein expression in cortical neurons by transfection of siRNAs directed against *PHD2* (Figure 3b). After 3 days in culture, the neurons were replated and allowed to grow processes for 24 h. Neurite elongation in cells transfected with *PHD2* siRNA was attenuated compared with the neurons transfected with control siRNA (Figure 3c). Furthermore, treatment with Y27632 completely blocked the inhibitory effect of silencing PHD2 gene expression (Figures 3c and d). These results show that PHD2 is crucial for neurite elongation by a mechanism dependent on the RhoA–Rho kinase pathway.

### Axonal sprouting from the CST after traumatic cortical injury is abrogated by PHD inhibition

To determine whether PHD inhibition alters the neurite elongation *in vivo*, we explored if PHD inhibitors suppressed axonal rewiring in the adult CNS following TBI. After a brain injury, the number of CST fibers that crossed the midline from the intact to the denervated side (i.e., recrossing fibers) is increased at the cervical level of the spinal cord following unilateral brain injury. These recrossing CST fibers make synapses with interneurons in circuits responsible for motor control, thereby contributing to compensation for impaired motor function. We thus asked whether inhibition of PHD signaling affected axonal rewiring after brain injury.

**Figure 4:**
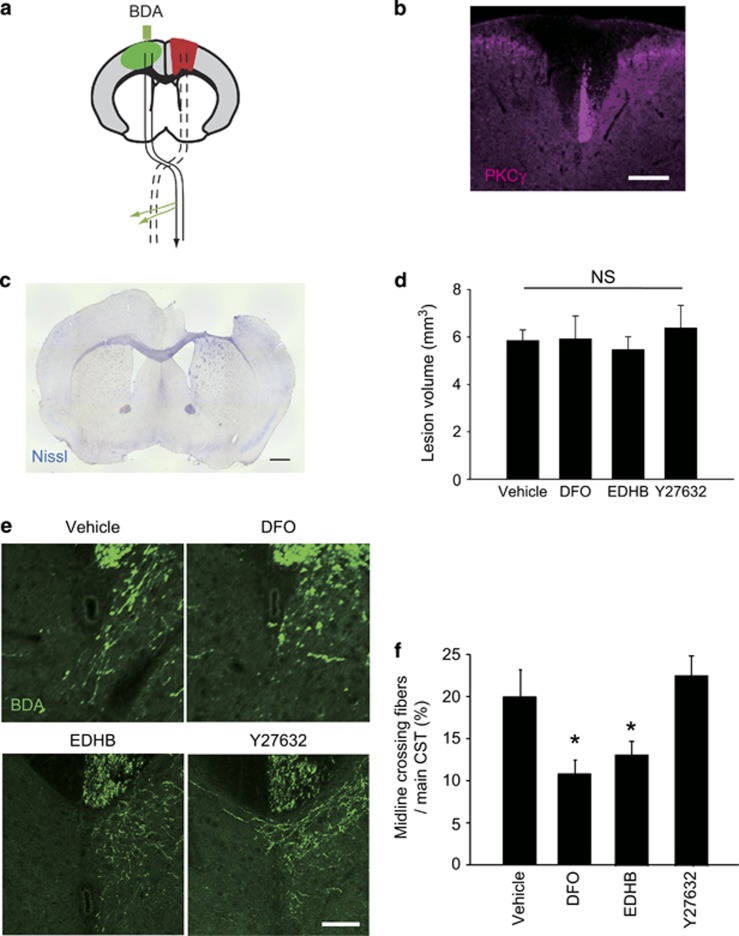
PHD inhibition suppresses the spontaneous reorganization of the corticospinal tract after traumatic brain injury. (**a**) Schematic illustration of corticospinal projections from the motor cortex and the experimental procedures. Traumatic injury to the sensorimotor cortex disrupted the CST (dotted line). BDA was injected into the contralesional motor cortex post procedure on the same day of the operation. The black arrow shows axons from the intact CST, which extends axonal branches sprouting and crossing the midline into the denervated side (green arrows). (**b**) Transverse sections of the spinal cord at the level of C6 level 4 weeks after the injury, showing destruction of CST in the dorsal columns stained for PKC*γ* (labeled with Alexa Fluor 568). (**c**) Representative Nissl-stained coronal section at the point of injury epicenter. (**d**) The graph shows mean±S.E.M. of lesion volume in mice treated for 4 weeks with indicated reagents (*n*=4-5). (**e**) Representative images of recrossing CST fibers labeled with BDA (labeled with Alexa Fluor 488) at the level of cervical cord 2 weeks after injury. (**f**) The graph shows mean±S.E.M. of number of the axons recrossing the midline in the cervical cord (*n*=3–5). *P<0.05 using Student's *t*-test. Scale bar, 200 *μ*m

First, we confirmed the destruction of the CST originating from the injured motor cortex after cortical impact (Figure 4a). The disappearance of CST from the injured motor cortex was confirmed by immunohistochemical assessment of protein kinase C (PKC)*γ* expression, a marker of the CST, in the cervical cord at 2 weeks after injury (Figure 4b). To test the role of PHDs in axonal rewiring, PHD inhibitors infused into contralateral motor cortex just after the injury and this was continued for 2 weeks. We observed no significant difference in the lesion volume between the control (saline) and PHD inhibitors-treated mice at day 14 after cortical injury, suggesting that PHD inhibition did not appear to affect injury pathology at the site of infarct (Figures 4c and d). To visualize the CST fibers, we simultaneously injected the anterograde tracer biotinylated dextran amine (BDA) into the motor cortex the day after injury. Two weeks after PHD inhibitors treatment, we counted the number of BDA-labeled CST fibers in the cervical spinal cord. The number of midline-crossing axons was significantly decreased in mice treated with DFO or EDHB, respectively (Figures 4e and f). Administration of Y27632 did not affect the number of midline-crossing axons compared with control (Figures 4e and f). These results indicate that the PHD inhibitor suppressed the innervation of contralateral corticospinal fibers into the affected CST tract after the traumatic brain injury.

### PHD inhibitor suppresses the spontaneous recovery of motor function after a traumatic brain injury

**Figure 5:**
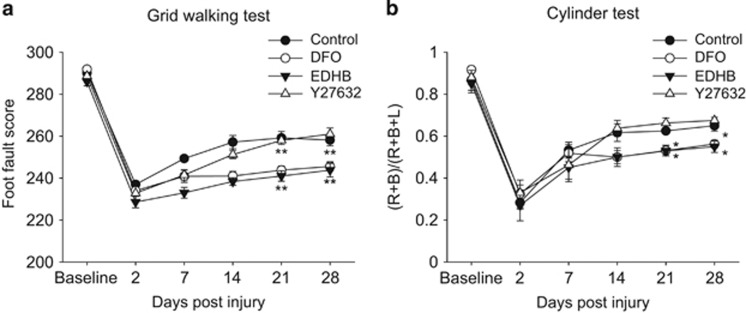
PHD inhibition prevents the recovery of motor function after a traumatic brain injury. Functional recovery of the impaired forelimbs in mice from the indicated groups. (**a**) Grid walk test. (**b**) Cylinder test. The graph shows mean±S.E.M. (*n*=4–9). **P<0.01, *P<0.05 using Bonferroni test

Finally, we investigated the effect of PHD inhibitor on functional recovery by two motor tests. The ability of mice to use their forelimbs was assessed with the cylinder test. Two days post injury, all mice showed deficits in using the contralesional right forelimb. Although mice in each group showed recovery of forelimb movement throughout the observation period, the behavioral performance was significantly worse in mice with PHD inhibitors treatment compared with control mice at 21 and 28 days after injury (Figure 5a). We also performed the grid walk test, which also estimates the function of the CST. Two days post injury, mice in both the groups showed marked deficits in their ability to accurately place the impaired forelimb. Again, mice treated with PHD inhibitors showed significant suppression of the spontaneous recovery of voluntary movement at 21 and 28 days, compared with mice treated with vehicle (Figure 5b). Furthermore, treatment with DFO during the initial 2 weeks led to sustained suppression of motor recovery over the full 4-week experimental period, suggesting that attenuation of PHDs may inhibit, but not delay, behavioral recovery after TBI.

## Discussion

Focal cortical injury results in neuroanatomical and neurophysiological changes in both adjacent and remote cortical tissue, as well as their subcortical fiber tracts. Because plastic changes in neural wiring are thought to contribute to the recovery of the cortical function, enhancing neural plasticity is a promising therapeutic strategy for patients with brain injury. Newly sprouting fibers from corticospinal tract form a compensatory network *via* propriospinal neurons, and thereby contribute to functional recovery.^1^ So far, enhancement of fiber sprouting from the corticofugal tract after brain injury has been accomplished in animal models by providing neurotrophic factors^18, 19^ and antibodies to axon growth inhibitors (NogoA),^20^ and modulating of neural activity.^21^ In the present study, we identified an additional candidate molecule, PHD, as an important regulator of axon rewiring after brain injury. We found that motor recovery was inhibited by treatment with a PHD inhibitor, which may be the result of diminished reorganization of adjacent axonal tracts into a compensatory neural network. Moreover, we observed widespread anatomical changes in the corticospinal axon following brain injury throughout the brain and spinal cord, suggesting that the effect of PHD inhibition on the spontaneous reorganization of the corticospinal tract might be observed at multiple levels of the CNS.

The severe limitation in the capacity for axon regeneration after an injury in the adult mammalian CNS is attributed to the presence of axon growth inhibitors at the lesion site. The majority of these inhibitory factors are components of CNS myelin and the glial scar (e.g., Nogo, myelin-associated glycoprotein (MAG), oligodendrocyte myelin glycoprotein (OMgp), chondroitin-sulfate proteoglycans (CSPGs), repulsive guidance molecule, and so on). Binding of many of these inhibitors to their neuronal receptors promotes the release of RhoA from Rho guanine dissociation inhibitor, resulting in activation of RhoA signaling cascades,^22^ and it is currently well accepted that inhibition of Rho facilitates axonal sprouting after a spinal cord injury (first evidence was provided by Dubreuil *et al.*).^23^ Thus, our findings that PHD inhibitors enhance protein expression of RhoA in cortical neurons and inhibit neurite elongation are in agreement with these previous findings and further indicate that PHD is an important, though previously unappreciated, regulator of RhoA signaling. Moreover, our observation that the effects of PHD inhibitors on neurite growth were abolished by the ROCK inhibitor demonstrates that the role of PHDs in axon regeneration is RhoA dependent.

Given that PHDs are essential modulators of HIF signaling and cellular oxygen-sensing mechanisms, it suggests that the hypoxia response may be important for axonal regeneration. Intracellular oxygen is mainly utilized for mitochondrial respiration. Current evidence indicates that the ATP production by mitochondria is required for axonal development, including growth cone motility, organelle transport, and cytoskeletal assembly.^24^ Intracellular localization of the three PHD family members has been previously reported; PHD1 localizes to the nucleus, while PHD2 and PHD3 are present in the cytoplasm.^25^ In addition, SM-20, the homologue of PHD3, is targeted via a specialized N-terminal motif to the mitochondria.^26^ SM-20 residing in the mitochondria is implicated in the control of cell growth and differentiation in skeletal muscle cells.^27, 28^ Although it is not clear if PHD2 associates with mitochondria, cell distortion in tumor vessels has been described in PHD2 haplodeficient mice.^29^ These previous observations support the possible involvement of mitochondrial activity in PHD2-regulated neuronal morphologenesis and neurite outgrowth.

Oxygen supply is modulated by changes in the tissue blood flow and the oxygen utilization, and/or neovascularization after injury. Neovascularization is considered to contribute in promoting the wound healing. Although its precise mechanism has not been elucidated fully, *in vivo* imaging data suggest a role for angiogenesis and neovascularization in axonal reorganization after a spinal cord injury.^30^ During the development of the peripheral nervous system, vasoconstrictors released from blood vessel acts as guidance cues for axons.^31^ In addition, we have previously reported that molecules produced by vascular endothelial cells also promotes neurite elongation in the CNS.^32, 33, 34, 35^ These findings suggest that axonal navigation is influenced by endothelium-derived factors. In this study, we observed that PHD is important for axon rewiring and functional recovery after a brain injury. Given that PHDs are an important for cellular oxygen sensing, they may integrate cues from the vascular system that regulate the process of axon remodeling and tissue repair after CNS injury. Future studies will explore the mechanistic basis of this potential relationship.

In summary, our results demonstrate that PHD enhances axonal reorganization and suggest that it may be a potential therapeutic target for the treatment of brain injury.

## Materials and Methods

### Mice

C57BL/6 J mice (SLC, http://www.jslc.co.jp/english/index2.htm) were bred and maintained in the Animal House of the Graduate School of Medicine, Osaka University. All experimental procedures were approved by the institutional committee of Osaka University.

### Primary culture of cortical neurons

Primary cultures of cortical neurons were obtained from cerebral cortex of postnatal day 1 (P1) mice. Cortices were triturated and dissociated with 0.25% trypsin in phosphate buffered saline (PBS) for 15 min at 37 °C. Trypsin was then inhibited by resuspension and trituration in Dulbecco's modified Eagle's medium (DMEM; Invitrogen, Carlsbad, CA, USA) containing 10% fetal bovine serum (FBS; MP Biomedical, Solon, OH, USA), followed by three washes in PBS. The isolated cells were plated at a density of 3 × 10^4^–4 × 10^4^ cells/cm^2^ into poly-l-lysine-coated dishes filled with DMEM containing 10% FBS and incubated for 24 h. To inhibit PHD, desferrioxamine mesylate salt (DFO; 100 *μ*M; Sigma-Aldrich, St. Louis, MO, USA) or ethyl-3,4-dihydroxybenzoate (EDHB; 100 *μ*M; Sigma-Aldrich) was added at the beginning of culture. Where indicated, cells were pretreated with Y27632 (10 *μ*M; Calbiochem, Darmstadt, Germany) for 10 min.

### Immunocytochemistry and neurite outgrowth analysis

The cells were fixed in 4% paraformaldehyde (PFA) in PBS for 30 min at 37 °C, and were permeabilized using 0.1% Triton-X-100 in PBS for 1 h at room temperature. After blocking with PBS containing 5% bovine serum albumin (BSA; minimum 98% electrophoresis grade, Sigma-Aldrich), cells were immunostained with monoclonal mouse anti-class III *β*-tubulin (TuJ1; 1 : 1,000; Covance, Madison, WI, USA; Cat# MMS-435 P-250, RRID: AB_10063408) and monoclonal anti-caspase 3 (1; 200; Cell Signaling, Danvers, MA, USA; Cat# 9668, RRID: AB_2069870) in PBS containing 5% BSA overnight at 4 °C. The samples were subsequently probed with mouse directed secondary antibodies for 2 h at room temperature and mounted onto coverslips using a fluorescent mounting medium (Dako, Carpinteria, CA, USA). The images were taken with an upright microscope (Olympus, Tokyo, Japan) and analyzed using a DP-controller image system (Olympus).

For the assessment of neurite length, we measured the longest neurite for each TuJ1–positive neuron and calculated the average of neurite length using ImageJ software (NIH). The effect of PHD inhibitors on cell survival was quantified by caspase 3 immunostaining. We estimated the ratio of caspase 3-positive neurons to total neurons.

### Western blot analysis

Cultured cells were lysed in 50 mM Tris-HCl, pH 8.0, 150 mM NaCl, 1% NP-40, 0.1% sodium dodecyl sulfate (SDS), 0.5% sodium deoxychorate, including protease inhibitor cocktail tablets (Roche Diagnostics, Burgess Hill, UK). The lysates were clarified by centrifugation at 15 000 r.p.m. at 4 °C for 10 min, and samples were normalized according to protein concentrations determined by the bicinchoninic acid protein assay (Pierce, Rockford, IL, USA). Proteins were separated by 10% SDS–polyacrylamide gel electrophoresis (SDS-PAGE) and transferred onto polyvinylidene difluoride membranes (Immobilon-P, Millipore, Bedford, MA, USA). Membranes were blocked for 1 h with 5% skim milk in PBS containing 0.05% Tween 20, incubated for 2 h with primary antibody, washed and incubated for 1 h with secondary antibody, and then visualized with the enhanced chemiluminescence (ECL) system (GE Healthcare, Waukesha, WI, USA). The following primary antibodies were used: mouse monoclonal anti-*α*-tubulin antibody (1 : 1000; Santa Cruz, Santa Cruz, CA, USA; Cat#sc-5286, RRID: AB_628411); mouse monoclonal anti-RhoA antibody (1 : 200; Santa Cruz, Cat# sc-166399, RRID: AB_2269522); rabbit monoclonal anti-PHD2 antibody (1 : 1000; Abcam, Cambridge, UK; Cat# ab109088, RRID: AB_10859674). Horseradish peroxidase-conjugated anti-mouse IgG and anti-rabbit IgG antibodies were used as secondary antibodies (1 : 5000; Cell Signaling).

### Preparation and transfection of small interfering RNA (siRNA)

Mouse PHD2 small interfering RNAs (siRNA) were purchased. The sense and antisense strands of *PHD2* siRNA (Stealth siRNA, Invitrogen) were as follows: 5′- GAGAUGGAAGAUGCGUGACAUGUAU -3′ (sense) and 5′- AUACAUGUCACGCAUCUUCCAUCUC -3′ (antisense). *PHD2* siRNAs were delivered into cortical neurons by using the Amaxa Nucleofector apparatus (program O-005). After 72 h *in vitro*, we have performed following experiments.

For analysis of neurite length in PHD2 siRNA-transfected neurons, cultured cells were collected by tripsynization and replated at a density of 3 × 10^4^–4 × 10^4^ cells/cm^2^ into poly-l-lysine-coated dished filled with DMEM containing 10% FBS and incubated for 24 h. In a separate experiment, we determined the knockdown efficiency by measuring PHD2 protein levels by using western blot analysis.

### Affinity precipitation of GTP-RhoA

Cortical neurons were treated with 100 *μ*M EDHB, 100 *μ*M DFO, or control medium for 5 min and then were lysed in a solution containing 50 mM Tris, pH 7.5, 1% Nonidet P-40, 5% glycerol, 1 mM Na_3_VO_4_, 1 mM NaF, 150 mM NaCl, 30 mM MgCl_2_, 1 mM DTT, and 10 *μ*g/ml each of leupeptin and aprotinin. The lysates were clarified by centrifugation at 15 000 r.p.m. at 4 °C for 10 min and surpernatants were incubated with 20 *μ*g of Rho-binding domain of rhotekin beads^15^ at 4 °C for 45 min. The beads were washed with lysis buffer and then performed to SDS-PAGE followed by immunoblotting with anti-RhoA antibody. The activation level was calculated by comparing the band intensities of active RhoA bands with those of total RhoA which was detected by immunobloting of cell lysate.

### Histology and immunohistochemistry

Animals were transcardially perfused with 4% PFA in PBS. Spinal cord and brain tissues were post-fixed with 4% PFA in PBS at 4 °C overnight, cryoprotected in 30% sucrose in PBS, and then embedded in optimal cutting temperature (OCT) compound (Tissue-Tek, Sakura Finetek, Torrance, CA, USA) for frozen sectioning. Coronal sections were cut at 30-*μ*m thickness on a cryostat and mounted on Matsunami adhesive silane-coated glass slides (Matsunami Glass, Osaka, Japan).

For histology, sections were stained with cresyl violet (Nissl stain; Sigma). Brain lesion volume was estimated by measurement of the area of lost tissue in each of three to five sections spaced 0.5 mm apart. The total lesion volume was calculated as described previously.^36^

For immunohistochemistry, sections were permeabilized in PBS containing 0.1% X-100 and 0.5% BSA for 1 h at room temperature. Sections were then incubated with primary antibodies overnight at 4 °C, followed by incubation with secondary antibodies for 2 h at room temperature. The primary antibodies used were as follows: mouse anti-NeuN (1 : 100; Merck, Cat# MAB377, RRID: AB_11210778), rabbit anti-PHD2 (1 : 200; Abcam), rabbit anti-PKC*γ* (1 : 100; Santa Cruz, Cat # sc-211, RRID: AB_632234). Alexa Fluor 488- and 588-conjugated goat anti-mouse IgG and goat anti-rabbit IgG antibodies (1 : 500; Invitrogen) were used as secondary antibodies.

### Traumatic cortical injury

Animals were stabilized in a stereotaxic frame (Muromachi Kikai). A midline incision was made in the scalp and the fascia was retracted to expose the cranium. A circular craniotomy opening of 4 mm diameter was performed with a drill on the left side, with the center at 0 mm anteroposterior and 2 mm lateral to the bregma. A sensorimotor controlled cortical impact was made by using a Pneumatic Impact Device (Amscien Instruments, Richmond, VA, USA) with a 3.0 mm flat-tip diameter, as described previously.^36^ The impact parameters were 4 mm/ms velocity, 1 mm depth, and 120 ms total time. The scalp was then sutured and closed and the mice were left to wake from the anesthesia.

### Anterograde labeling of the CST

To visualize the CST, the anterograde tracer biotinylated dextran amine (BDA; 10 000 MW; dilution, 10% in PBS; Invitrogen) was slowly injected with a glass capillary attached to a micro-syringe into the forelimb motor area as determined by a functional map of the motor cortex (coordinates from bregma: 0 mm anterior/1.0 mm lateral, 0.5 mm anterior/1.0 mm lateral, 0 mm anterior/1.5 mm lateral, and 0.5 mm anterior/1.5 mm lateral, 0.4 *μ*l per site, all at a depth of 0.5 mm into cortex).^37^

Two weeks after BDA injection, we obtained transverse cryosections of spinal cord from C4 to C6. For visualization of BDA-labeled fibers, the sections were treated with 0.3% Triton-X-100 in PBS for 1 h, followed by incubation with Alexa Fluor 488-conjugated streptavidin (1 : 400; Invitrogen) in PBS for 2 h. To quantify the number of crossing axons, we counted the number of BDA-positive fibers crossing from the intact side of the spinal cord to the contralateral side in 10–20 sections for each mouse. To normalize for differences in the tracing efficiency of the individual animals, the number of fibers crossing the midline was divided by the total number of labeled main CST fibers in the dorsal column.

### Pharmacological treatment

A 28-gauge stainless steel cannula attached to a plastic pedestal (Brain infusion kit 3, Alzet, Cupertino, CA, USA) was introduced through a burr hole in the skull and into the forelimb area of the right motor cortex (coordinates from bregma: 1.0 mm anterior, 1.0 mm lateral, and 0.65 mm depth from the cortical surface). The cannula was cemented to the skull by using cyanoacrylate and connected via plastic tubing to a subcutaneously implanted Alzet osmotic pump (Model 1002, Alzet). The pumps were filled with vehicle solution (saline), EDHB (55.04 *μ*g/kg/day over 2 weeks), DFO (197.36 *μ*g/kg/day over 2 weeks), or Y27632 (9.021 *μ*g/kg/day over 2 weeks) dissolved in saline.

### Grid walk test

The grid-walking test assesses the ability to accurately place the forepaws on the rungs of a grid during spontaneous exploration.^18, 38, 39^ Mice were placed on a wire grid (200 × 240 mm) with 12-mm square holes and allowed to freely explore for 3 min. Performance was recorded with a video camera. In the first 50 steps, the number of footslips of the impaired right forepaw was assessed. A footslip was scored using a Foot Fault Scoring System.^40^ Pretraining on the grid-walking test is not necessary, but each animal was tested once before surgery to obtain a baseline score.

### Cylinder test

The cylinder test evaluates forelimb use during spontaneous vertical exploration within a cylinder.^41^ Mice were placed in a cylinder (9 cm in diameter and 15 cm in height) and recorded with a video camera. We counted the number of contacts with the cylinder wall made during a full rear movement by the left and right forelimbs independently and by both forelimbs simultaneously. During a rear movement, if one forelimb (e.g., the right forelimb) made several contacts or dropped immediately after simultaneous contact, the movement was scored twice as ‘both' and ‘left'. A total of 20 movements were recorded. Cylinder test performance was scored as: (impaired right forelimb use+both forelimbs use)/total use. The cylinder test does not require pretraining, but was performed once by each animal before surgery to obtain a baseline score. The mice which showed inability of performing the tasks were excluded the analysis.

### Statistical analysis

Data are presented as mean±S.E.M. For behavioral scores, significance between groups was examined using the Mann–Whitney *U* test. Other statistics were analyzed by using either an unpaired Student's *t-*test or one-way analysis of variance test (ANOVA) followed by Scheffe's tests or Tukey's tests. *P-*values <0.05 were considered to be significant.

## Abbreviations

ANOVA: analysis of variance
BDA: biotinylated dextran amine
BSA: bovine serum albumin
CNS: central nervous system
CSPG: chondroitin-sulfate proteoglycans
CST: corticospinal tract
DFO: desferrioxamine
DMEM: Dulbecco's modified Eagle's medium
ECL: enhanced chemiluminescence
EDHB: ethyl-3,4-dihydroxybenzoate
FBS: fetal bovine serum
HIF: Hypoxia Inducible Factors
MAG: myelin-associated glycoprotein
OCT: optimal cutting temperature
OMg: poligodendrocyte myelin glycoprotein
P: postnatal day
PAGE: polyacrylamide gel electrophoresis
PBS: phosphate buffered saline
PFA: paraformaldehyde
PHD: Prolyl 4-hydroxylases
PKC: protein kinase C
ROCK: Rho-associated protein kinase
SDS: sodium dodecyl sulfate
TBI: traumatic brain injury
Tuj1: class III *β*-tubulin
